# Equilibrial service composition model in Cloud manufacturing (ESCM) based on non-cooperative and cooperative game theory for healthcare service equipping

**DOI:** 10.7717/peerj-cs.410

**Published:** 2021-03-01

**Authors:** Ehsan Vaziri Goudarzi, Mahmoud Houshmand, Omid Fatahi Valilai, Vahidreza Ghezavati, Shahrooz Bamdad

**Affiliations:** 1 School of Industrial Engineering, South Tehran Branch, Islamic Azad University, Tehran, Tehran, Iran; 2 Department of Industrial Engineering, Sharif University of Technology, Tehran, Tehran, Iran; 3 Department of Mathematics & Logistics, Jacobs University Bremen, Bremen, Bremen, Germany

**Keywords:** Cloud manufacturing, Service composition, Industry 4.0, Game theory

## Abstract

Industry 4.0 is the digitalization of the manufacturing systems based on Information and Communication Technologies (ICT) for developing a manufacturing system to gain efficiency and improve productivity. Cloud Manufacturing (CM) is a paradigm of Industry 4.0. Cloud Manufacturing System (CMS) considers anything as a service. The end product is developed based on the service composition in the CMS according to consumers’ needs. Also, composite services are developed based on the interaction of MCS providers from different geographical locations. Therefore, the appropriate Manufacturing Cloud Service (MCS) composition is an important problem based on the real-world conditions in CMS. The game theory studies the mathematical model development based on interactions between MCS providers according to real-world conditions. This research develops an Equilibrial Service Composition Model in Cloud Manufacturing (ESCM) based on game theory. MCS providers and consumers get benefits mutually based on ESCM. MCS providers are players in the game. The payoff function is developed based on a profit function. Also, the game strategies are the levels of Quality of Service (QoS) based on consumers’ needs in ESCM. Firstly, the article develops a composite service based on a non-cooperative game. The Nash equilibrium point demonstrates the QoS value of composite service and the payoff value for the players. Secondly, the article develops a composite service based on a cooperative game. The players participate in coalitions to develop the composite service based on formal cooperation. The grand coalition demonstrates the QoS value of composite service and the payoff value for the players in the cooperative game. The research has compared the games’ results. The players’ payoff and the QoS value are better in the cooperative game than in the non-cooperative game. Therefore, the MCS providers and consumers are satisfied mutually in the cooperative game based on ESCM. Finally, the article has applied ESCM in a Healthcare Service to equip 24 hospitals in the best time.

## Introduction

After the beginning of the Age of Reason, Adam Smith and Charles Darwin expressed the division of labor in their books. In the 1750s, in the Pins Factory example, Smith referred to the subject of specialization. He stated that wealth generation is increased by dividing labor into labor subdivisions and making it more efficient by using specialized skills. It seems that the most significant improvement in the power of production is affected by the division of labor. Based on the concept of Natural Selection and Survival of the Fittest in Darwin's book, Origin of Species, and the Free Market issue in Smith's book, Wealth of Nations, the rational individuals and companies may selfishly create more profits for themselves and the system (Siegfried, 2006). Today, Industry 4.0 (also called the Fourth Industrial Revolution) changes the traditional pyramid model of automation to a model of interconnected services through connecting systems and sharing data to gain efficiency and improve productivity, generating an ecosystem among clients, suppliers, and producers. This change is described as disruptive and has already been implemented in large companies (Velásquez, Estevez & Pesado, 2019). A remarkable transformation that emerges with Industry 4.0 is the shift from centralized to decentralized control for reaching a highly flexible production of customized products and services (Dallasega et al., 2020). Cloud Manufacturing (CM) is a paradigm of Industry 4.0. Cloud Manufacturing System (CMS) reduce the cost and increase the utilization rate of resources. A CM research project was launched in Europe in 2010, sponsored by the European Commission. The goal of this project is to provide users with the ability to utilize the manufacturing capabilities of configurable and virtualized production networks. Specialized and customized demands can be better satisfied based on the flexible and fast-reaction nature of a CMS (Wang & Xu, 2013b). Manufacturing Cloud Service (MCS) is the self-contained, configurable, and on-demand manufacturing service package comprise of the manufacturing resources and manufacturing capabilities for fulfilling consumer’s needs based on the CMS (Wang & Xu, 2013b; Delaram & Valilai, 2018a; Valizadeh et al., 2019; Valizadeh, Fatahi Valilai & Houshmand, 2020). The problem of Optimal Service Selection and Composition is categorized among the hardest problems in combinatorial optimization as an NP-hard problem (Fontanini & Ferreira, 2014; Jula, Sundararajan & Othman, 2014; Li, Yao & Zhou, 2016; Liu et al., 2017; Zhou & Yao, 2017c). Game theory is recommended to solve the problem in the prior researches. Also, the researchers less have investigated developing a mathematical model for MCS composition to mutually satisfy expectations of consumers and service providers based on the game theory in CMS.

The motivation for this article is to answer two questions simultaneously in a CMS. The first question is: “How to develop services with high levels of Quality of Service (QoS) based on the consumers’ considerations?” The second question is: “How to develop services that are most beneficial to the service providers?” Therefore, the main motivation of the article is to satisfy the high benefit of consumers and service providers mutually as an equilibrium state in the CMS based on real-world conditions. The research proposes the cooperation between MCS providers as a solution to satisfy the motivation. So, the article proposes a solution to develop cooperation between MCS providers for increasing the MCS benefit and the level of QoS mutually based on real-world conditions and game theory. The main contribution of the article is developing a mathematical model for MCS composition in the equilibrium state. The research has developed an Equilibrial Service Composition Model in Cloud Manufacturing (ESCM) based on game theory. Firstly, the article develops a composite service based on a non-cooperative game. MCS providers participate in developing the composite service as the game players. The players select the QoS levels as the game strategies, selfishly. Also, the QoS levels are determined based on the consumers’ needs. The article proposes a function for determining the players’ payoff. Therefore, the Nash equilibrium point demonstrates the QoS value of composite service and the payoff value in the non-cooperative game. Secondly, the article develops a composite service based on a cooperative game. The players participate in coalitions to develop the composite service based on formal cooperation. The grand coalition demonstrates the QoS value of composite service and the payoff value for the players in the cooperative game. The payoff value and QoS value are better in the cooperative game than the non-cooperative game based on games’ results. Therefore, the MCS providers and consumers get benefit mutually in the cooperative game in ESCM. The results show the QoS value and MCS providers’ payoff value based on the classical manufacturing system are less than the values based on ESCM. Finally, the article has applied the ESCM in a healthcare service as a case to equipping 24 hospitals with three service providers in the best time at two provinces in a country. The equipping time is reduced based on the results in ESCM.

## Literature Review

### Cloud Manufacturing

**Figure 1:**
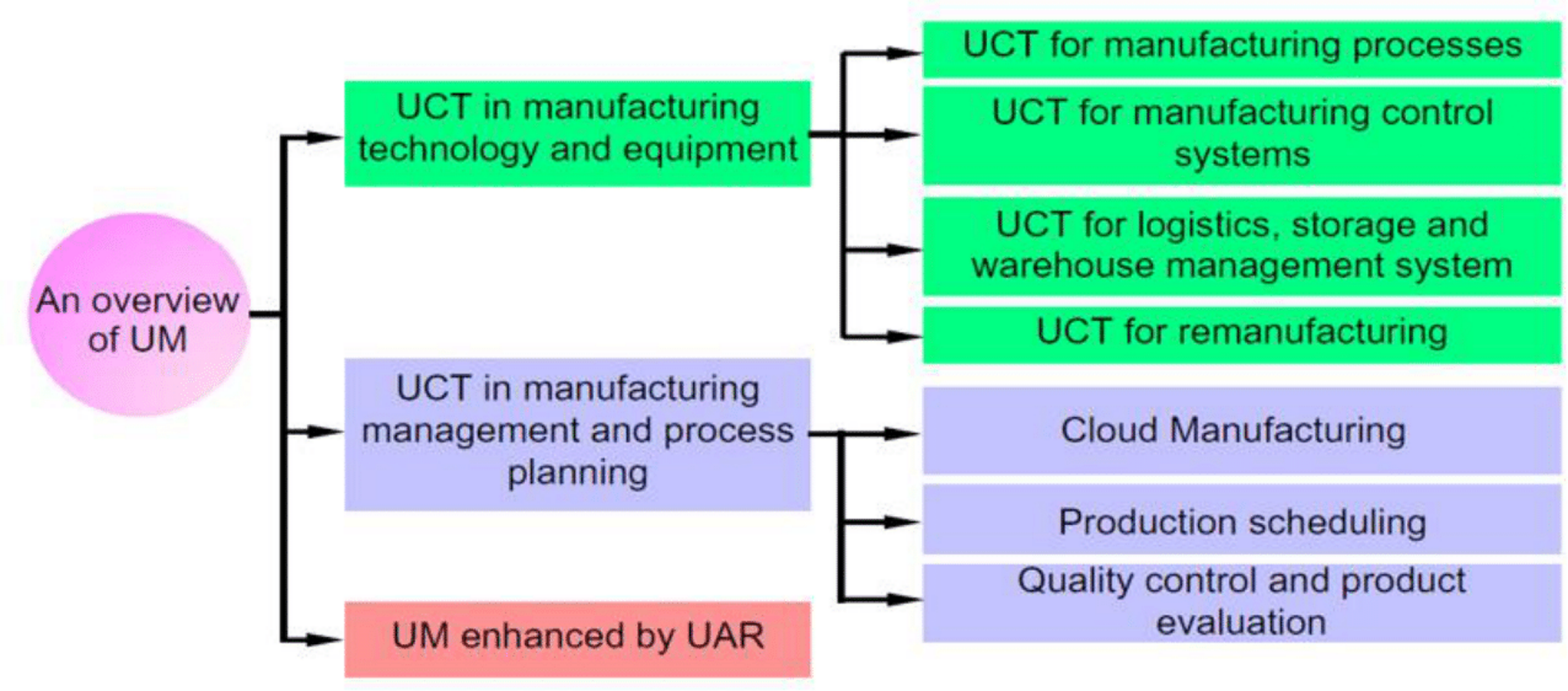
An overview of ubiquitous manufacturing (Wang, Ong & Nee, 2017) (UCT: Ubiquitous Computing Technology, UAR: Ubiquitous Augmented Reality). Reprinted with permission of Taylor & Francis. ©Taylor & Francis.

Cloud Manufacturing is one of the in-depth concepts that few studies discuss (Liu et al., 2019). CM is changing the future perspective of the manufacturing industry by moving from traditional production-oriented manufacturing to service-oriented manufacturing (Bouzary, Chen & Krishnaiyer, 2018; Fatahi Valilai & Houshmand, 2015). The CM paradigm proposes an efficient approach in which distributed manufacturing resources are encapsulated into cloud services and managed in a centralized way (Fatahi Valilai & Houshmand, 2015). Industry 4.0 and CM constitute the two significant efforts of taking advantage of information technologies to promote the further development of the manufacturing industry in the manufacturing community (Liu & Xu, 2017). Also, CM has been conducted by researchers as a subset of Ubiquitous Manufacturing (UM) and a paradigm of Industry 4.0. CM topic is expressed by researchers between 2012 and 2017. Also, researchers’ attention has increased to the UM topic because the topic addresses a wide range of subjects related to the manufacturing industry. Figure 1 proposes the UM application based on three categories. CM is a core technology in UM. In a CMS, the allocation of production resources and inventories are applied as manufacturing services in a pay-as-you-go model, for example, Design-as-a-Service and Hardware-as-a-Service (Wang, Ong & Nee, 2017).

### Manufacturing Cloud service composition

One of the most critical issues to improve CM is Manufacturing Service Management (MSM) based on QoS. Service Composition and Optimal Selection are essential in MSM (Tao et al., 2015; Assari, Delaram & Valilai, 2018). When a single cloud service (i.e., a software image and a virtual machine) cannot satisfy all the consumer requirements, service composition is required (Dastjerdi & Buyya, 2014) based on QoS (Wang & Xu, 2013a; Delaram & Valilai, 2018b; Aghamohammadzadeh, Malek & Valilai, 2019). Service composition is a field of an interoperable solution for CM research in the service community (Mietzner et al., 2010). MCS composition is a process that the fundamental issue of providing on-demand manufacturing services in the cloud is the mapping of distributed manufacturing resources with personalized service requests (Lu & Xu, 2017). MCS composition means taking advantage of current virtual services to provide a new service that does not exist on its own to execute a complex task that a single service cannot do (Guo et al., 2010).

The QoS measures are cost, time, availability, reliability, and reputation (Jula, Sundararajan & Othman, 2014; Wu et al., 2013b; Zhou & Yao, 2017c). The QoS levels are considered based on the service level agreement to define consumers’ needs for the performance of composite services, such as price and response time (Wang et al., 2016a). One of the most critical issues for improving the CMS and QoS is the Optimal Service Selection and Composition (Tao et al., 2015; Aghamohammadzadeh & Valilai, 2020). Selecting and combining MCSs into a composite service to meet the consumer’s requirements, while keeping up the optimal service performances, is of paramount importance in CM (Bouzary, Chen & Krishnaiyer, 2018). The distribution of cloud service providers demonstrates a geographically dispersive manner (Valilai & Houshmand, 2014), which elevates the impact of the network on QoS in CMS (Wang, Yang & Mi, 2015). According to the distribution of the Manufacturing Cloud Services (MCSs) in different geographic areas (Wang, Yang & Mi, 2015) and the massive similar manufacturing functionalities and a variety of QoS (Wang, Ong & Nee, 2017), the Optimal Service Selection and Composition is an important, complex and complicated problem in the CM (Jula, Sundararajan & Othman, 2014; Wang, Yang & Mi, 2015; Wang, Ong & Nee, 2017; Delaram & Valilai, 2016, 2017).

**Figure 2:**
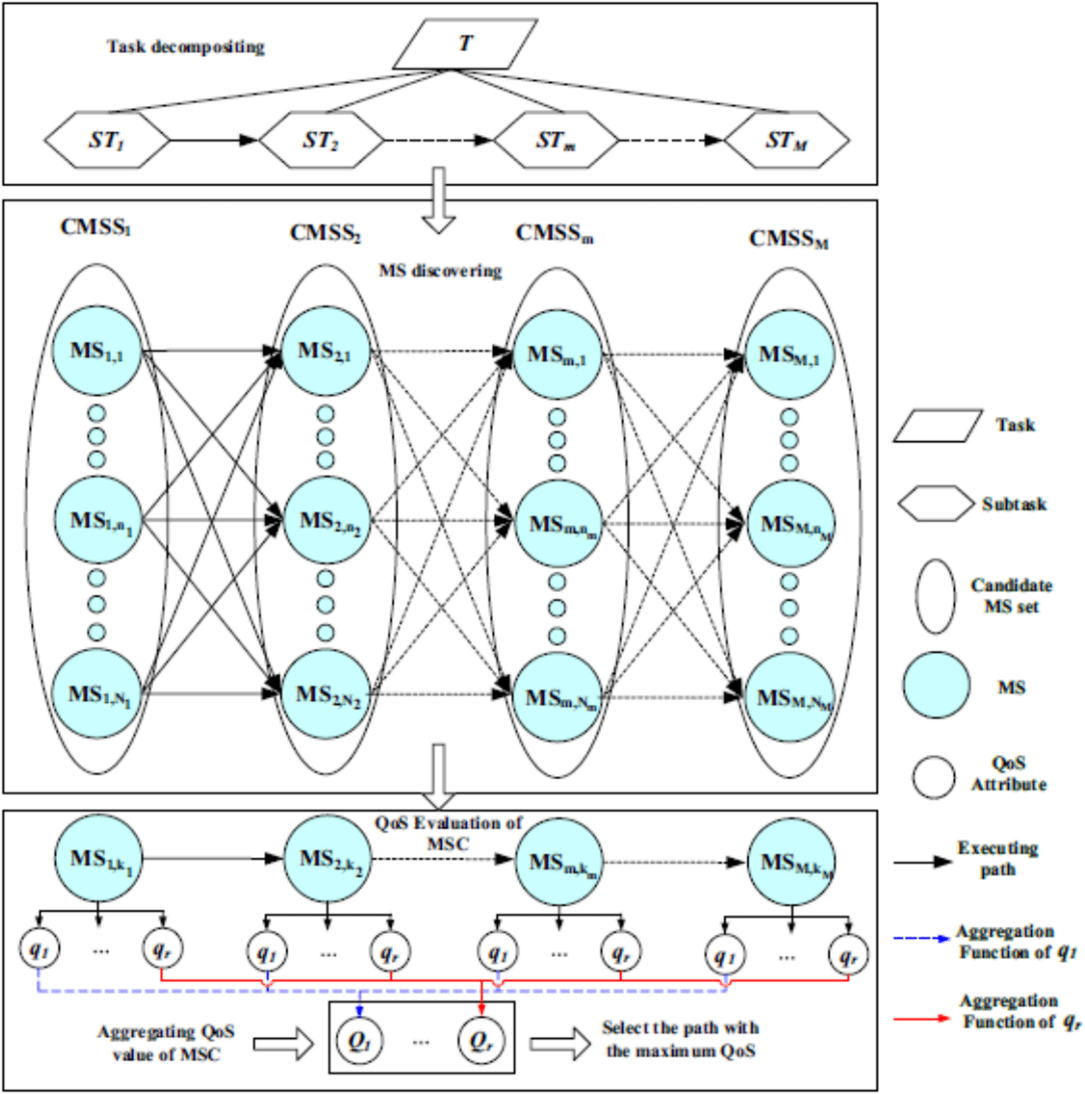
Conceptual overview of the MSC architecture (Zhou & Yao, 2017a) (MS: Manufacturing Service, STs: Subtasks, CMSS: Candidate MS Set, MSC: Manufacturing Service Composition). Reprinted with permission of Springer Nature. ©Springer Nature.

Zhou & Yao (2017a) have proposed an overview of a manufacturing service composition in Fig. 2. The processes of composition are Task decomposition, Manufacturing service discovering, and Manufacturing service composition. The task decomposition process state that the CM platform decomposes the consumers’ task into subtasks. In the manufacturing service discovering process, the CM platform tries for finding qualified services for assigning to the subtask according to requirements and then generates the related candidate manufacturing service set. The manufacturing service composition process selects a service from the candidate service set for developing ways of manufacturing service composition. Also, the manufacturing service composition process selects one path with the optimal overall performance and the appropriate QoS based on the consumers’ requirements (Zhou & Yao, 2017a).

### Cloud service broker

It is difficult for service consumers to choose the most suitable service provider. Also, it is difficult, especially for the service providers, to provide the best service at the negotiated price, while the service providers and the service consumers themselves can’t automatically negotiate. Service consumers usually cannot negotiate, manage, and monitor the QoS. As a third-party mediation between service consumer and service provider, CSB is responsible for processing the consumer’s requests (Chen et al., 2016). CSBs sit between the cloud service consumer and the cloud service provider to manage the discovery of the service, intermediation, security, and governance (Linthicum, 2017). Wu et al. (2013a) mentioned the Cloud-Based Design and Manufacture model that is composed of a cloud consumer, cloud provider, CSB, and cloud carriers. In the model, the CSB is an intermediate party between the consumers and providers. The CSB manages the use, performance, and delivery of services in the model.

### Game theory

In 1950, Isac Asimov published his famous science-fiction novel, Foundation Trilogy. The hero of the novel as a mathematician, Hari Seldon, has a plan called Psychohistory to control the human based on a mathematical system. The Psychohistory has developed based on the thought of Romans and Greeks about the nature of human behavior called the Code of Nature. By entering the Age of Reason in the eighteenth century, some scientific including Adam Smith in his book, Wealth of Nations, proceeded to discover the Code of Nature as a key to understanding the natural discipline of human interactions. Smith has tried to find out a discipline similar to the Physics Gravity Law for economic and social behaviors. Also, Psychohistory enables Seldon to predict social, economic, and political desires in the Foundation Trilogy. Game theory is the key to achieve that discipline. At the same time as Asimov, John Nash published an article expressing the game theory principles. Indeed, modern game theory was emerged based on John von Neumann’s article in 1928. Therefore, Nash has expanded the game theory scope. Also, he reinforced game theory to solve real-world problems as Newton and Einstein’s Mathematics is at the service for Physics problems. Accordingly, Nash’s Mathematics provides service for the social and biological sciences. Game theory is the strategy science and tells us what choices are most useful when interacting with others (Siegfried, 2006).

Game theory is used for modeling decision-makers’ behaviors in strategic situations. Since the structures of games are different, the behavior and preferences of the players are different in various types of games. Each game contains three main elements (Sadigh, Chaharsooghi & Sheikhmohammady, 2016): (1) Players as the set of decision-makers, (2) Strategy refers to the player’s choices, (3) Payoff is the specified value for each player in every combination of strategies. There have been two kinds of researches in game theory: Cooperative games theory pioneered by Von Neumann and Morgenstern in 1953 and Non-cooperative games theory developed by Nash in 1951. Even when the players, the set of strategies, and payoffs table of a cooperative game are all the same as that of a non-cooperative game, one player may choose different strategies and receive different payoffs in the two games. Rational players will choose strategies to make the game to be cooperative or non-cooperative for maximizing their payoffs (Li, 2004).

Game theory is a theoretical tool for analyzing cooperative behavior or conflict between different individuals. Game theory can play an essential role in manufacturing service management. The service composition process chooses several services as a candidate to create a final composite service. So, different businesses cooperate to fulfill a requested service in the process. The companies cooperate or compete with each other based on different conditions. Game theory is used to design an appropriate service providing algorithm. Also, game theory takes into account the interest of various companies and can create a beneficial communication environment (Tao et al., 2015).

Jula, Sundararajan & Othman (2014), Wang et al. (2016b), Liu & Zhang (2017), Zheng, Feng & Tan (2016), Henzel & Herzwurm (2018), Adamson et al. (2017), Goodarzi et al. (2020) and Zhang & Hu (2015) have emphasized developing an appropriate model for MCS composition based on the QoS. Also, Tao et al. (2015), Wu, Schaefer & Rosen (2013c), Fontanini & Ferreira (2014), Li et al. (2012) and Zeng, Zhao & Zeng (2009) have recommended using game theory for solving the MCS composition problem in CM. The article has investigated the prior researches related to the MCS composition problem, as shown in Table 1. Fontanini & Ferreira (2014) have modeled the interaction between suppliers as players in a non-cooperative and incomplete information game. The suppliers know only their utility function in the model (Fontanini & Ferreira, 2014). Wahab et al. (2016) have distinguished two types of service providers: leaders and followers in a Stackelberg game. Leaders are providers that possess a high reputation whereas followers are those providers that cannot compete against the leaders (Wahab et al., 2016). Esposito et al. (2016) have developed a non-cooperative game. The game players are selfish without direct communication between them. The player only tries to minimize its own cost without considering the state of the other players (Esposito et al., 2016). Lei & Junxing (2017) have modeled the interactions of agents to propose a service composition method based on reinforcement algorithm and game theory. The agents as players interact with other agents in a cooperative state to learn and select the strategies (Lei & Junxing, 2017). Li et al. (2012) have modeled the competition of services as a non-cooperative game. The model analyzes the competitive relationships between tasks as players (Li et al., 2012). Zhang & Hu (2015) have established a model for logistics cloud services discovery and combination based on game theory. Chen et al. (2016) have developed an incomplete information dynamic game between a service provider and a service consumer. The research focuses on how people transmit information through an oral or written statement in private information and information asymmetry situations (Chen et al., 2016).

**Table 1:** The related dominant *literature* studies on ESCM.

Research	MCS composition	Using game theory	Cooperative game based on core coalition
Fontanini & Ferreira (2014)	Yes	Yes	No
Wahab et al. (2016)	Yes	Yes	No
Esposito et al. (2016)	Yes	Yes	No
Lei & Junxing (2017)	Yes	Yes	No (Learning Algorithm)
Li et al. (2012)	Yes	Yes	No
Zhang & Hu (2015)	Yes	Yes	No (Virtual Enterprise Alliance)
Li, Yao & Zhou (2016)	Yes	No (Promoted GA)	No
Qiu (2014)	Yes	No	No
Sasikaladevi (2016)	Yes	No (Knapsack)	No
Wang, Yang & Mi (2015)	Yes	No (Novel algorithm via graph theory)	No
Wang et al. (2016a)	Yes	No (GA and annealing)	No
Huang, Liu & Duan (2014)	Yes	No (MCOP)	No
Dastjerdi & Buyya (2014)	Yes	No (Fuzzy)	No
Wang et al. (2016b)	Yes	No (Promoted MIP)	No
Huang et al. (2015)	Yes	No (Proposed algorithm SCA)	No
Li et al. (2016)	Yes	No (Proposed algorithm BEA)	No
Liu & Zhang (2017)	Yes	No (SESG-SC based on GA)	No
Liu et al. (2017)	Yes	No (Proposed algorithm based on MMAS)	No
Xu et al. (2016)	Yes	No (Bee algorithm based on pareto optimal)	No
Zheng, Feng & Tan (2016)	Yes	No (PSO and Fuzzy)	No
Liu et al. (2016)	Yes	No (Normalization via SAW)	No
Zhou & Yao (2017c)	Yes	No (Proposed HABC algorithm)	No
Zhou & Yao (2017a)	Yes	No (Proposed algorithm based on artificial bee colony)	No
Zhou & Yao (2017e)	Yes	No (Proposed HABC algorithm based on bee colony)	No
Zhou & Yao (2017d)	Yes	No (Proposed HABC algorithm based on bee colony)	No
Zhou & Yao (2017f)	Yes	No (Proposed MPsaDABC Algorithm)	No
Zhou & Yao (2017b)	Yes	No (Proposed HABC algorithm based on bee colony)	No
Chen et al. (2016)	Yes	Yes	No
This research	Yes	Yes	Yes

### Study of related researches to the ESCM

The article has studied the previous researches to investigate the ESCM. Some studies have used game theory for service composition in CMS. So, the article has categorized and shown the related researches to the ESCM based on game types in Table 1.

## Materials and Methods

### Equilibrial service composition model in Cloud manufacturing

This article has developed a mathematical model for solving the MCS composition problem in the equilibrium state based on game theory. The model develops non-cooperative and cooperative games to compose MCSs based on the consumers’ needs to obtain the equilibrium state. The equilibrial state generates appropriate profit for manufacturers and the appropriate QoS.

The game players are MCS providers. The game strategies are the QoS levels based on the consumers’ needs. The game payoff is the earned value based on the players’ selected strategies. So, the game payoff is determined based on the proposed profit function in the research. In the non-cooperative game, MCS providers compete to choose an appropriate strategy to participate in composite service selfishly. The Nash Equilibrium (NE) point determines the QoS level. Also, the payoff value is determined based on the QoS level. Therefore, the total payoff value is divided equally between the players in the non-cooperative game. In the cooperative game, MCS providers cooperate in developing the composite service based on a formal agreement. Therefore, the players generate coalitions to develop the composite service. The grand coalition is the stable core in the cooperative game. The core shows the QoS level in the composite service. According to the comparison of results, the players’ payoff in the cooperative game is better than the non-cooperative game according to the results. Also, the QoS value in the cooperative game is better than the non-cooperative game. The comparison shows the importance of cooperation between MCS service providers in CMS.

### The games structure in ESCM

This research supposes only one consumer order in ESCM. Also, the players have similar preferences to develop the composite service in the game. Two games are formed as non-cooperative and cooperative games. There are three players and three QoS levels as the players’ strategies in each game. The highest profit is allocated to the highest QoS level in the strategies. The players are selected from one or more Manufacturing Clouds (MCs) according to consumer order. The players choose strategies according to their organizational conditions and the service development cost. Therefore, the strategic form of the game is shown in Eq. (1).

(1) $$\matrix{ {{\text{Players}};} & {N = \left\{ {A,B,C} \right\}} \cr {{\text{Players Strategies;}}} & {{S_A} = \left\{ {{q_i}} \right\},\>{S_B} = \left\{ {{q_i}} \right\},\>{S_C} = \left\{ {{q_i}} \right\};\>{q_i} \in \left\{ {QoS} \right\}} \cr {{\text{Strategy Profile;}}} & {S = {S_A} \times {S_B} \times {S_C}} \cr {{\text{Payoff function;}}} & {U\left( S \right) = {u_i}} \cr } $$

The game payoff for each player is determined based on the player strategy and the other players’ strategies. The article develops a mathematical profit function $P({q_i})$ in Eq. (2) based on a proposed function by Abdoli (2011). The profit function is defined based on the cooperation between players to develop the composite service. There are two cooperation types in the ESCM. Firstly, the amount of services is distributed between the players. In the first type, each player develops the complete service based on the referred amount of services. The end QoS value determines based on the arithmetic mean of QoS values. Secondly, processes of service development are distributed between the players based on the specialized subjects of the processes. The end QoS value determines based on the weighted average of QoS values. The research considers the first type of cooperation between the players. This type of cooperation is a consortium between players to participate in service composition. According to the Holonic approach in CMS, the research results can be generalized to the second type of cooperation between MCS providers.

(2) $$P\left( {{q_i}} \right) = k\left( {\displaystyle{{{q_1} + {q_2} + {q_3}} \over 3} + c{q_1}{q_2}{q_3}} \right);\; {q_i} \in \left\{ {QoS} \right\};\; 0 < c \le \displaystyle{1 \over 9}$$

The article develops the payoff function based on the profit function, as shown in Eq. (3).

(3) $${U_i}({q_i}) = \displaystyle{P \over 3} - {q_i^2}\Rightarrow {U_i}({q_i}) = \displaystyle{k \over 3}\left(\displaystyle{{{q_1} + {q_2} + {q_3}} \over 3} + c{q_1}{q_2}{q_3}\right) - {q_i^2};\; 0 < c \le \displaystyle{1 \over 9};\; 0 < {q_i} \le 4$$

The above terms are: ${U_i}$ as the Payoff function for each player, ${q_i}$ as the selected strategy of player *i*, $P\left( {{q_i}} \right)$ as the Profit function, ${q_i^2}$ as the cost of service provided based on the QoS level *i*, *c* as the completeness degree between all players, and *k* as the profitability coefficient based on the proposed QoS.

### The non-cooperative game in ESCM

In the non-cooperative game, there is no formal contract between the players to cooperate. Also, the players are rational to select the best strategies selfishly. Therefore, the players are aware of the other players’ selfishness and rationality. So, the players choose the best strategy to maximize their expected payoff based on their perception of the other players’ strategies. The best response of players is developed based on the best responses of other players. So, the system of linear equations is developed for determining the best response of each player and the Nash equilibrium point in the game, as shown in Eq. (4).

(4) $$\eqalign{& {u_1}({q_1},{q^*_2},{q^*_3}) = \displaystyle{k \over 3}\left(\displaystyle{{{q_1} + {q^*_2} + {q^*_3}} \over 3} + c{q_1}{q^*_2}{q^*_3}\right) - {q^2_1} \cr & {u_2}({q^*_1},{q_2},{q^*_3}) = \displaystyle{k \over 3}\left(\displaystyle{{{q^*_1} + {q_2} + {q^*_3}} \over 3} + c{q^*_1}{q_2}{q^*_3}\right) - {q^2_2} \cr & {u_3}({q^*_1},{q^*_2},{q_3}) = \displaystyle{k \over 3}\left(\displaystyle{{{q^*_1} + {q^*_2} + {q_3}} \over 3} + c{q^*_1}{q^*_2}{q_3}\right) - {q^2_3}}$$

After equations differentiation based on the variables of QoS value (${q_i}$) in the system of linear equations, the new system of linear equations is determined and shown in Eq. (5).

(5) $$\eqalign{& \displaystyle{{\partial {u_1}} \over {\partial {q_1}}} = \displaystyle{k \over 9} + \displaystyle{{kc} \over 3}{q^*_2}{q^*_3} - 2{q^*_1} = 0 \cr & \displaystyle{{\partial {u_2}} \over {\partial {q_2}}} = \displaystyle{k \over 9} + \displaystyle{{kc} \over 3}{q^*_1}{q^*_3} - 2{q^*_2} = 0 \cr & \displaystyle{{\partial {u_3}} \over {\partial {q_3}}} = \displaystyle{k \over 9} + \displaystyle{{kc} \over 3}{q^*_1}{q^*_2} - 2{q^*_3} = 0}$$

Therefore, the best response of players (${q_i^*}$) is determined after solving the new system of linear equations, as shown in Eq. (6).

(6) $$\eqalign{ \displaystyle{k \over 9} + \displaystyle{{kc} \over 3}{q^*_2}{q^*_3} = 2{q^*_1} \cr  \displaystyle{k \over 9} + \displaystyle{{kc} \over 3}{q^*_1}{q^*_3} = 2{q^*_2} \cr  \displaystyle{k \over 9} + \displaystyle{{kc} \over 3}{q^*_1}{q^*_2} = 2{q^*_3}}$$

The Nash equilibrium point is determined based on Eq. (6) in the Non-cooperative game, as shown in Eq. (7).

(7) $${q^*_1} = {q^*_2} = {q^*_3} = - \displaystyle{{\sqrt {36 - \displaystyle{{4c{k^2}} \over 3}} - 6} \over {2ck}}\quad {\rm or}\quad \displaystyle{{\sqrt {36 - \displaystyle{{4c{k^2}} \over 3}} + 6} \over {2ck}}$$

By supposing $k = 6$ and $c = \displaystyle{1 \over 9}$, the Nash equilibrium point is $(0.4,0.4,0.4)$ based on the first answer. Also, the second answer is impossible according to the ${q_i}$ acceptable range in Eq. (3). Therefore, the expected payoff of players is 0.7, as shown in Eq. (8).

(8) $${u_i}\left( - \displaystyle{{\sqrt {36 - \displaystyle{{4c{k^2}} \over 3}} - 6} \over {2ck}}, - \displaystyle{{\sqrt {36 - \displaystyle{{4c{k^2}} \over 3}} - 6} \over {2ck}}, - \displaystyle{{\sqrt {36 - \displaystyle{{4c{k^2}} \over 3}} - 6} \over {2ck}}\right) = 0.7$$

The players don’t assure the other players’ cooperation in the Non-cooperative game. Therefore, the players select the lower level of the QoS as the strategy in the Nash equilibrium point. So, the players obtain low payoff value in the non-cooperative game.

### The cooperative game in ESCM

The players should participate in three types of coalitions based on the formal contract in the cooperative game. (1) The players can participate in the game as a single-player. The players compete based on a non-cooperative game in the first type of coalition. (2) The players can participate in a coalition of two players as the second coalition type. Two players generate the coalition. Therefore, the coalition competes with the third player based on a non-cooperative game in the second type of coalition. (3) The players can participate in a coalition of three players as the grand coalition. The players generate the grand coalition to develop the composite service based on a cooperative game in the third type of coalition.

The characteristic function determines the players’ payoff according to the coalitions, as shown in Eq. (9). The value of the characteristic function is determined based on the game matrix form in the first and second types of coalitions.

(9) $$\matrix{ {V(\left\{ A \right\}) = 1.2} & {V(\left\{ {A,B} \right\}) = 2.4}  {}  \cr    {V(\left\{ B \right\}) = 1.2}  & {V(\left\{ {A,C} \right\}) = 2.4} & {V(\left\{ {A,B,C} \right\}) = 18.7}  \cr    {V(\left\{ C \right\}) = 1.2} &  {V(\left\{ {B,C} \right\}) = 2.4}  {}  \cr  }$$

The system of linear equations is developed based on the characteristic function and the core stability requirements, as shown in Eq. (10). In the equation, ${X_i}$ is the players’ expected payoff based on the characteristic function. The payoff value of the grand coalition is a higher value than other coalition’s payoff value based on the stability requirements. Therefore, the grand coalition is a stable core solution based on the core stability requirements in the cooperative game.

(10) $$\matrix{{{X_1} + {X_2} + {X_3} = 18.7} & {{\rm{(Grand Coalition (Core) Equation}})}  \cr {{X_1} + {X_2} \ge 2.4} & {}  \cr {{X_2} + {X_3} \ge 2.4} & {}  \cr {{X_1} + {X_3} \ge 2.4} & {{\rm{(Core Stability Conditions)}}}  \cr {{X_1} \ge 1.2} & {}  \cr {{X_2} \ge 1.2} & {}  \cr {{X_3} \ge 1.2} & {}  \cr } $$

After solving the system of equations, the core stability requirements are determined in Eq. (11). Therefore, the core is stable based on the stability requirements.

(11) $$\eqalign{ 1.2 \le {X_1} \le 16.3 \cr  1.2 \le {X_2} \le 16.3 \cr  1.2 \le {X_3} \le 16.3}$$

Also, the research has determined the exact value of payoff ${X_i}$ based on the Shapley value solution. The Shapley value solution was introduced by Shapley (1953). The solution determines the Shapley value as the player payoff value, as shown in Eq. (12).

(12) $${{\rm \varphi} _i}(V) = \sum\limits_{\scriptstyle S \subset N \atop \scriptstyle i \in S} ^{} {\displaystyle{{(\left| S \right.\left| { - 1} \right.)!(n - \left| S \right.\left| ) \right.!} \over {n!}}} \left[ {V(S) - V(S - \left\{ i \right\})} \right]$$

The above terms are: ${{\rm \varphi} _i}(V)$ as the assigned value to the player *i*, $\left| S \right.\left| {} \right.$ as the number of members in the coalition, *n* as the number of game players, $\left[ {V(S) - V(S - \left\{ i \right\})} \right]$ as the coalition value after the player *i* joins the coalition.

The research supposes the impact values of players are equal in the coalitions. Therefore, the players’ Shapley values are similar. The players’ payoff is shown in Eq. (13) based on the Shapley values in the cooperative game.

(13) $${{\rm \varphi} _i} = ({{\rm \varphi} _1},{{\rm \varphi} _2},{{\rm \varphi} _3}) = (6.23,6.23,6.23)$$

According to the cooperative game result, the MCS providers as the players choose the highest level of QoS as the game strategy based on the grand coalition in the cooperative game. Also, the players’ payoff is the highest value.

## Results

### Study of ESCM in the healthcare services

**Figure 3:**
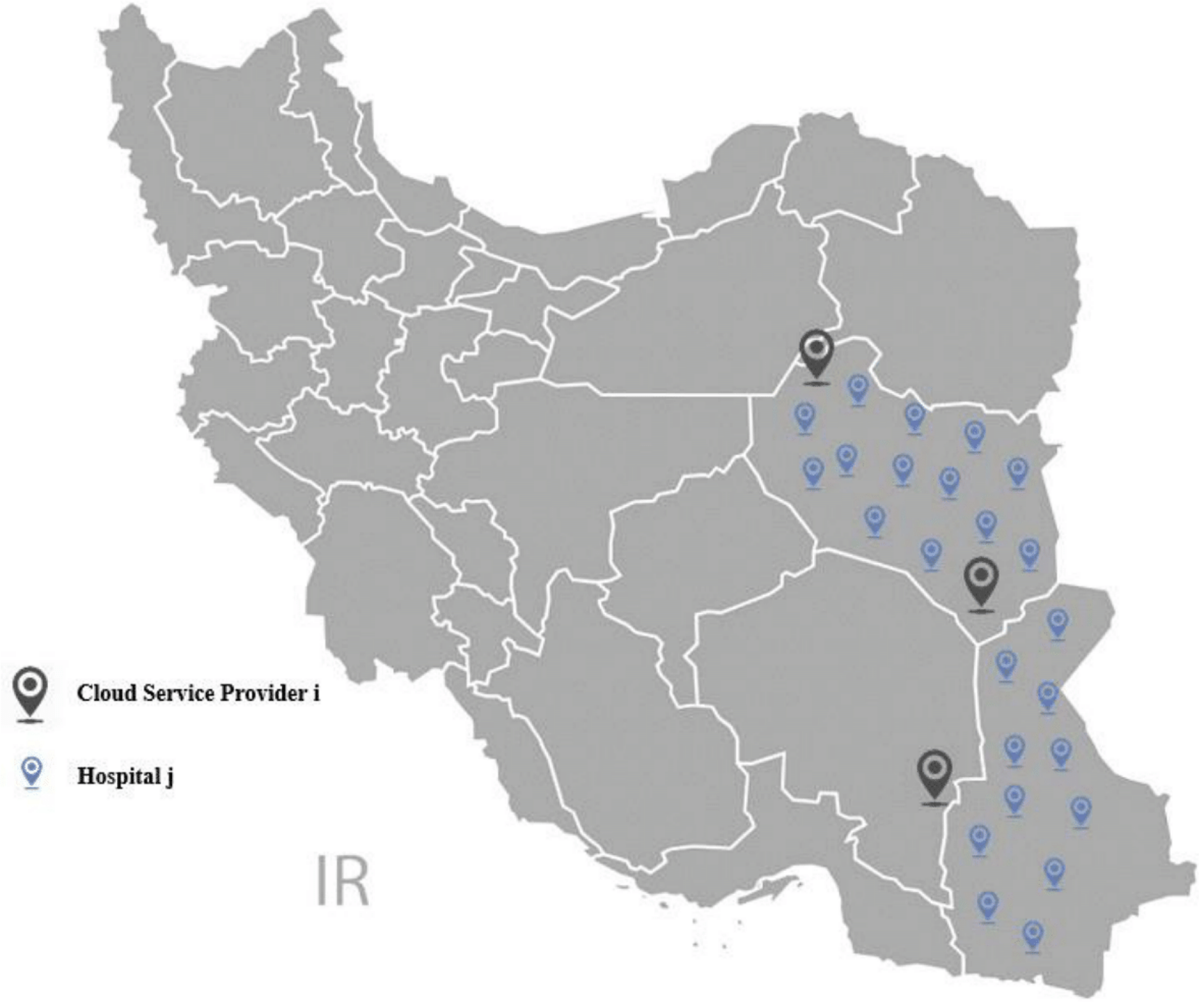
Distribution map of the hospitals and companies in the case study.

The research applies ESCM for equipping the hospitals in two provinces of Iran. Twenty four hospitals are considered from different provinces to equip with Medical Ultrasound devices. Also, there are some distribution companies to install the devices in the hospitals and to train hospital staff on how to work with the device, as shown in Fig. 3. It is very time-consuming to perform the whole project by only one company. Also, the company carries out the device establishment for each hospital in 2 days. This process may take over 2 days for some cities based on the distance between the city and the company. Therefore, transportation costs will increase according to the distances.

**Figure 4:**
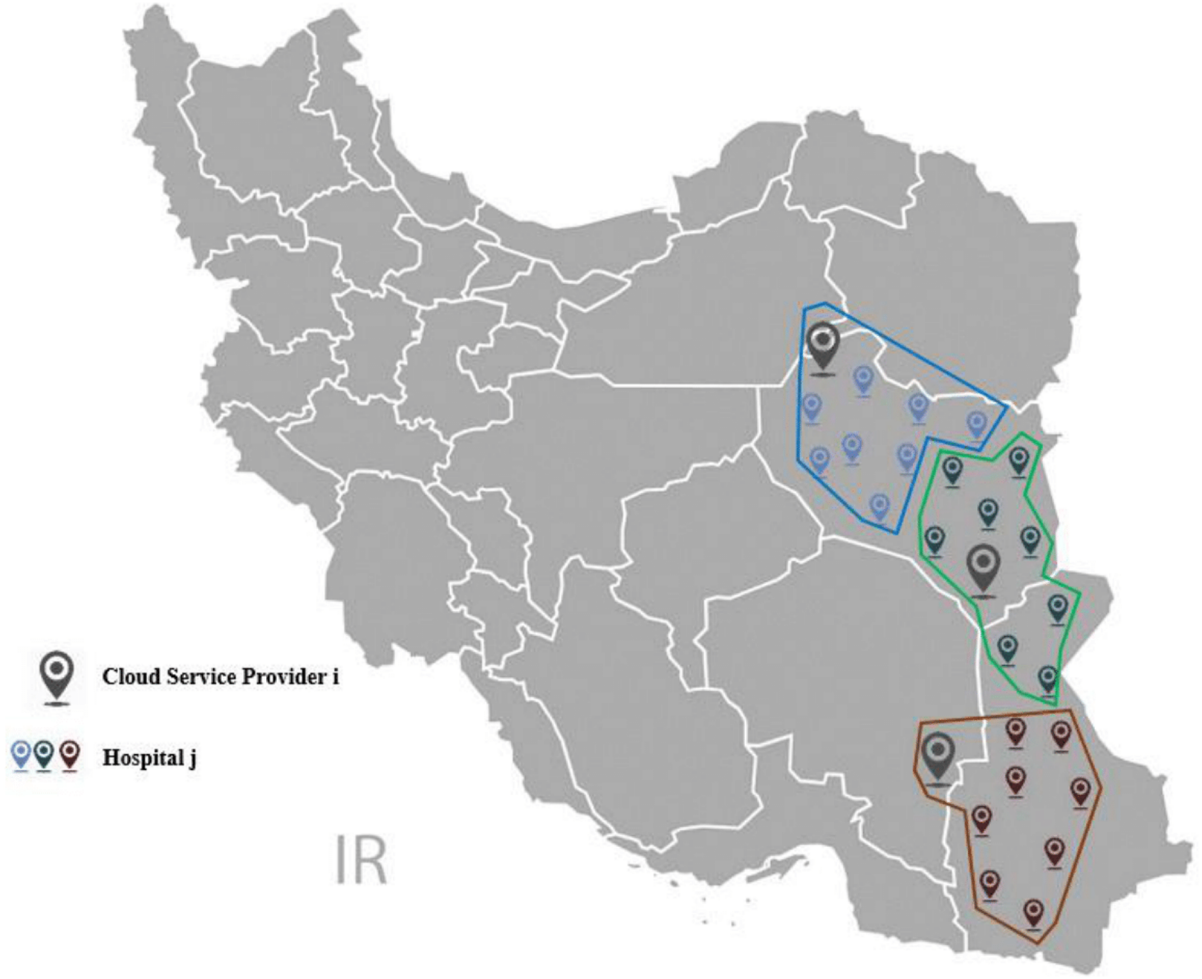
Clustered map of the hospitals and companies as the cloud service providers.

The research applies ESCM to improve the work process in the case study. The companies are considered as cloud service providers in ESCM. So, three cloud service providers are selected according to the geographical location of the hospitals. These service providers perform the device establishment in the appropriate reliability state. Eight hospitals are assigned to each cloud service provider based on the geographical location, as shown in Fig. 4. Each cloud service provider assigns between one and four teams to establish the devices in the hospitals.

In this case study, the healthcare management organization and the CSB have signed a formal contract with each other. The healthcare management pays the service cost to CSB based on the QoS levels. The total QoS value is determined based on the average of the QoS values provided by cloud service providers. Finally, CSB has divided the service revenue between cloud service providers.

The article supposes that cloud service providers supply services with constant reliability in the appropriate state. Also, the service cost is determined based on the service time. So, the QoS measure is the service time in the case study. Therefore, the cloud service system revenue is determined based on the service time. Four service levels are demonstrated based on the QoS in the case study:

First level: QoS value = 4, each cloud service providers assign 4 teams to service, *t* = 4 (days).

Second level: QoS value = 3, each cloud service providers assign 3 teams to service, *t* = 6 (days).

Third level: QoS value = 2, each cloud service providers assign 2 teams to service, *t* = 8 (days).

Fourth level: QoS value = 1, each cloud service providers assign 1 team to service, *t* = 16 (days).

The research develops the game based on the ESCM in the case study. The game players are cloud service providers. The game strategies are the four levels of QoS. The game payoff is the revenue of cloud service providers.

The research considers *k* = 9 and *c* = 1/7 in the case study, according to ESCM. Accordingly, the results of the cooperative and non-cooperative games are shown in Table 2 based on ESCM.

**Table 2:** The games results in the case study.

Game type	The QoS value (q_i_*)	The payoff (*u_i_*)
Non-cooperative game	0.6	1.5
Cooperative game	4	23.3

The game players select the fourth level in the non-cooperative game. So, the hospitals are equipped in 16 days by three cloud service providers. However, the game players cooperate in the stable grand coalition to select the first level in the cooperative game. So, the hospitals are equipped in 4 days by three cloud service providers in the cooperative game. According to the importance of processing time for equipping the hospital, the research suggests applying the ESCM in the cooperative game type for satisfying the healthcare management in the best service time.

## Discussion

Most small and medium manufacturers can provide services such as machining, drilling, CAD, CAM, etc. The manufacturers are not capable of performing large projects alone. Therefore, the manufacturers don’t achieve the high value-added of the projects. However, large manufacturing companies develop all manufacturing processes in the companies or based on the particular supply chain. CMS develops virtualized factories to satisfy the large manufacturing projects based on the small and medium manufacturers as the MCS providers. The service cost, time, and reliability, as the QoS measures, are improved by utilizing the small and medium manufacturers as the MCS providers in CMS. Also, CMS will replace the new MCS providers to improve system resilience, whether the MCS provider is inaccessible. The end service is developed based on the service composition in CMS.

MCS Composition problem studies service development through MCS providers based on the consumers' needs. The problem should consider the expectations of MCS providers and consumers’ needs based on real-world conditions mutually. Therefore, this research use game theory for solving the MCS Composition problem. The main novelty of the article is to propose a model for considering the expectations of the consumers and the service providers simultaneously based on the game theory in CMS. The essential expectation of consumers is a high level of QoS. Also, the essential expectation of service providers is a high value of profit. The end service is developed by various MCS providers in the CMS. Therefore, all service providers should get a high value of profit mutually. The model considers the expectations of consumers as game strategies in the model. The consumers determine the level of QoS as the strategies. Also, the service providers are considered as the players in the model. The selected strategies by players determine the players’ payoff value and the QoS level mutually. Therefore, both types of expectations are distinguished by players based on QoS levels in the model. The article develops two types of models based on game theory. The first type is developed based on the non-cooperative game. The player payoff value is determined based on the selected QoS levels by all players. The payoff value of a player has decreased whether the player selects a higher level of QoS than the other players’ selected QoS levels in the non-cooperative game. Thus, the players select the low level of QoS as strategies selfishly to decrease the cost of service providing in the non-cooperative game. Therefore, the expectations of service providers and consumers are fulfilled at a low level based on the Nash equilibrium point in the game. The first game type tries to demonstrate real-world conditions in CMS. The research has established cooperation between the service providers as players based on the cooperative game type in the second type of model. The players cooperate in a grand coalition to develop the composite service. All players select a high level of QoS to improve the benefit of the coalition and to increase the payoff value in the cooperative game. Thus, the expectations of service providers and consumers are satisfied at a high level in the second model simultaneously. Finally, the research represents that the expected payoff value and the QoS level are increased in the cooperative game toward the non-cooperative game. Therefore, the article recommends the cooperative game model as a solution to satisfy the consumers and service providers mutually based on real-world conditions.

## Conclusion

The research has proposed ESCM as the model for the MCS composition problem based on game theory. The game players are MCS providers that compete for participating in the composite service based on the cooperative and non-cooperative games in the ESCM. The game strategies are the QoS levels based on the consumer’s needs. The end product is developed based on a composition of several smaller MCS in the CMS. Therefore, the earned income is divided equally among the MCS providers in ESCM. Thus, MCS providers selfishly reduce the QoS level in a non-cooperative game to reduce their production costs. The Nash equilibrium point proposes a low level of QoS in the non-cooperative game. So, the end product has been provided to the consumers at the lowest level of QoS. Also, MCS providers get a low payoff in the game. Developing cooperation between the MCS providers is proposed as a solution to mutual increase the benefit of MCS providers and the level of QoS by this research. The article provides a solution to develop cooperation between MCS providers based on the cooperation game and real-world conditions. In the cooperative game, the players cooperate based on the formal contract. The players have selected the highest level of QoS as the strategy in the cooperative game. Accordingly, the end product has been provided to the consumers at the highest level of QoS. Also, the players have gotten a high payoff based on the core solution and the Shapley value method in the grand coalition. Therefore, the MCS providers and consumers are satisfied mutually based on ESCM in the cooperative game. Finally, the article has applied ESCM to equip 24 hospitals in a healthcare service as a case study. Based on the results, developing formal cooperation between the service providers reduces the service time and increases the benefit of the service providers mutually. Therefore, service providers and consumers are satisfied mutually.

Future research should consider the Blockchain-based Smart Contract for formal cooperation between MCS providers based on real-world conditions. Also, future research should develop a model of MCS composition based on specific reliability function and infinitive MCS providers as the players in the game.

## Additional Information and Declarations

### Data Availability

The following information was supplied regarding data availability: The data set is available at Figshare: Fatahi Valilai, Omid (2020): Hospitals in South Khorasan-Iran. figshare. Dataset. DOI 10.6084/m9.figshare.13143767.v2. Codes are also available at Figshare: Fatahi Valilai, Omid (2020): Service composition Codes. figshare. Software. DOI 10.6084/m9.figshare.13173224.v1.
